# Depsipeptides Targeting Tumor Cells: Milestones from In Vitro to Clinical Trials

**DOI:** 10.3390/molecules28020670

**Published:** 2023-01-09

**Authors:** Plinio A. Trinidad-Calderón, Carlos Daniel Varela-Chinchilla, Silverio García-Lara

**Affiliations:** Tecnológico de Monterrey, School of Engineering, and Sciences, Avenida Eugenio Garza Sada 2501, Monterrey 64849, Nuevo León, Mexico

**Keywords:** depsipeptide, anticancer, cancer-targeting, cell, functional food, enrichment

## Abstract

Cancer is currently considered one of the most threatening diseases worldwide. Diet could be one of the factors that can be enhanced to comprehensively address a cancer patient’s condition. Unfortunately, most molecules capable of targeting cancer cells are found in uncommon food sources. Among them, depsipeptides have emerged as one of the most reliable choices for cancer treatment. These cyclic amino acid oligomers, with one or more subunits replaced by a hydroxylated carboxylic acid resulting in one lactone bond in a core ring, have broadly proven their cancer-targeting efficacy, some even reaching clinical trials and being commercialized as “anticancer” drugs. This review aimed to describe these depsipeptides, their reported amino acid sequences, determined structure, and the specific mechanism by which they target tumor cells including apoptosis, oncosis, and elastase inhibition, among others. Furthermore, we have delved into state-of-the-art in vivo and clinical trials, current methods for purification and synthesis, and the recognized disadvantages of these molecules. The information collated in this review can help researchers decide whether these molecules should be incorporated into functional foods in the near future.

## 1. Introduction

Nowadays, cancer is considered the first or second major cause of death in multiple countries around the world [1,2]. In 2020, more than 19 million new cases and nearly 10 million deaths associated with this disease were reported [3,4,5]. Unsurprisingly, cancer cases are expected to reach the 24 million mark by 2035, and their incidence to triple by 2050 [6,7].

Although recognized cancer therapies are being further enhanced [8], the consumption of enriched foods to boost the recovery of cancer patients is also gaining attention [9]. To date, nearly 35% of chemotherapeutic drugs are nature-derived products, making these an important source of oncological pharmaceuticals [10]. Nonetheless, only a limited number of recognized molecules found in foods, especially in animals, have been shown to possess promising cancer cell-targeting abilities in preclinical trials [11].

**Figure 1:**
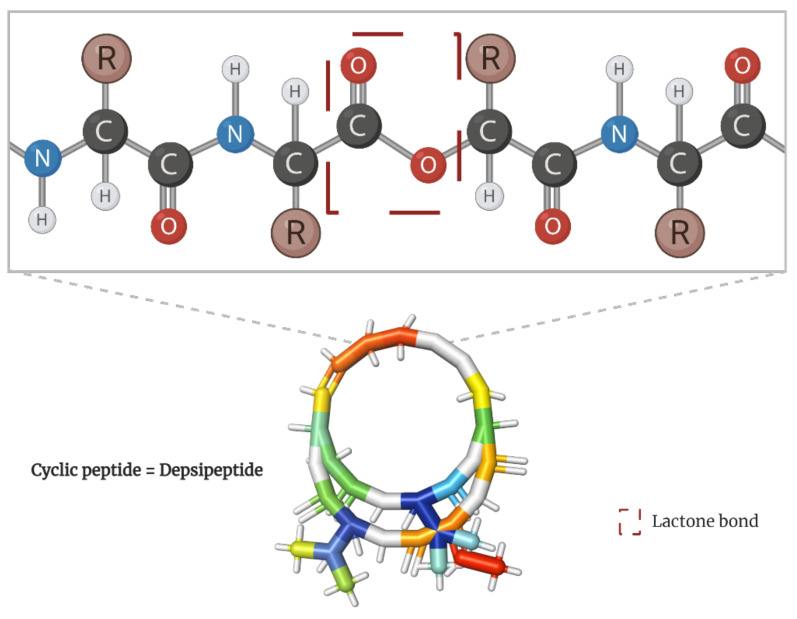
Peptide bond structure of depsipeptides. Lactone bond in the core ring marked in red.

Among the spectrum of potential therapeutic molecules, the tumor-targeting capability of natural cyclic peptides has been deeply explored [12,13]. Scientifically known as depsipeptides, these peptide cyclo-oligomers are characterized by having one or more amino acids replaced by a hydroxylated carboxylic acid, resulting in one lactone bond in a core ring (Figure 1) [14]. Interestingly, they account for more than 1300 natural molecules reported to contain different acyl groups and other moieties [15,16] as well as to perform varied bioactivities [17,18].

Hitherto, few authors have recognized that the vast majority of depsipeptides are found in marine species, most of which are not present in easily accessible areas and are hard to reproduce in the laboratory environment without proper instruction [10,19]. Some other authors have highlighted that the knowledge on “anticancer” mechanisms, known limitations, and faced challenges of using these molecules is minimal [20].

In response, here, we have comprehensively reviewed specific depsipeptides with known sequences, elucidated their molecular structures, and recognized cell death mechanisms whereby they target tumor cells. Moreover, we have discussed the state-of-the-art in vivo milestones, current methods of synthesis and encapsulation, their advancement in clinical trials, and the principal disadvantages of tumor-targeting depsipeptides to determine if they can be considered as serious candidates for cancer therapeutics, and ultimately, enrich functional foods.

### Methodology Used in Literature Research

We searched both Scopus and the Google Scholar database using the keywords “depsipeptide”, “cancer”, and “targeting”. Both original research and review articles that were chosen for analysis were published no earlier than 2017. The articles with the keywords “antibody”, “conjugate”, “fraction”, “proteomic”, “saccharide”, and “vaccine” were ineligible for this review.

## 2. Depsipeptides with a Recognized Mechanism of Targeting Cancer Cells

Depsipeptides induce cytotoxicity by various mechanisms, with their principal features being the capacity to perforate cell membranes and tubulin–microtubule imbalance, eventually inducing apoptosis [21]. In this section, we classified depsipeptides based on the mechanisms underlying their targeting cancer effects.

### 2.1. Depsipeptides Inducing Apoptosis in Tumor Cells

Apoptosis, as the most prevalent and well-defined form of programmed cell death [22], plays an important role in regulating tumor cells [23]. Unsurprisingly, resistance to apoptosis—due to the inactivation or loss of caspases [24] and dysregulation of the mitochondrial pathway [25]—is considered a major advantage for oncogenic cells [26].

**Figure 2:**
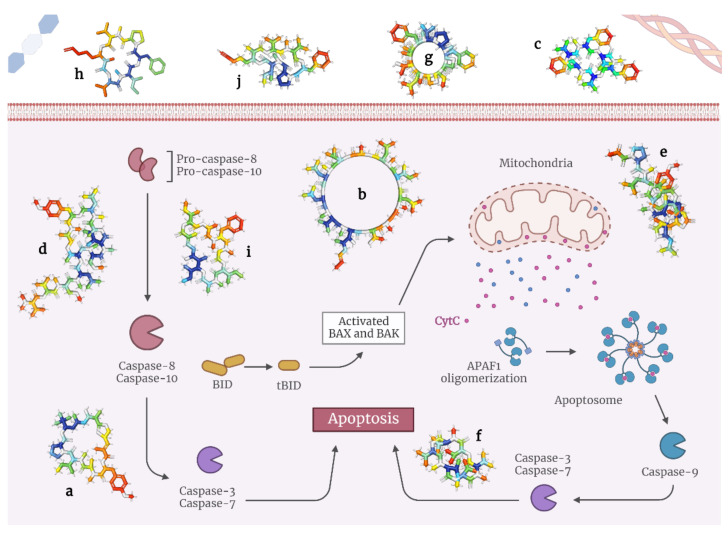
Models of depsipeptides inducing apoptosis in tumor cells. (**a**) Apratoxin A; (**b**) Aurilides; (**c**) Beauvericins; (**d**) Dehydrodidemnin B; (**e**) Enniatins; (**f**) Lagunamides; (**g**) Coibamide A; (**h**) Grassypeptolides; (**i**) Hantupeptins; (**j**) Tiahuramides.

In general, these depsipeptides potently activate caspase-3, Fas/FasL, p21, and p27, and inhibit histone deacetylases, all of which result in pro-apoptotic signaling [27,28,29,30] Here, we present the state-of-the-art on depsipeptides that induce apoptosis in tumor cells (Table 1 and Figure 2).

#### 2.1.1. Apratoxin A

Originally isolated from multiple species of the genus *Lyngbya*, apratoxins are cyclic depsipeptides composed of peptides and polyketide fragments with known cytotoxic activity [21]. Several apratoxins have been recognized to date and studied in vivo (Section 3.1) [31,40,41,42].

Specifically, apratoxin A dose-dependently activates caspase-3 and caspase-7, and eventually induce caspase-dependent apoptosis as well as autophagy in the absence of endoplasmic reticulum stress [29]. Additionally, this depsipeptide is cytotoxic toward human epithelial carcinoma (KB) and colorectal cancer (LoVo) cell lines [43].

Apratoxin A also regulates the transcription of cell cycle genes, inducing the G_1_ phase arrest and subsequent apoptosis of HT29 colon cancer cells [44]. Moreover, it inhibits the phosphorylation of signal transducer and activator of transcription 3, thereby inhibiting fibroblast growth factor signaling and angiogenesis [45].

Recently, apratoxin S10, a novel apratoxin analog that inhibits the growth of two pancreatic cell lines (EC46 and EC68) by inhibiting receptor tyrosine kinases, vascular endothelial growth factor A (VEGF-A), and interleukin-6 [31], has also demonstrated potent inhibition of highly vascularized cell models including hepatocellular carcinoma (Huh-7), renal cell carcinoma (A-498), and neuroendocrine cancer (NCI-H727) [46].

#### 2.1.2. Aurilides

These cyclic depsipeptides, comprising a macrocyclic carbon skeleton and six amino acid-derived moieties [21], were originally derived from the sea slug *Dolabella auricularia*. Aurilides are reported to be potently cytotoxic toward murine neural crest-derived cancer cells (Neuro-2a), a cervical cancer cell line (HeLa), and NCI-60 cell lines, a cell panel created by the U.S. National Cancer Institute to characterize the genomic markers of drug sensitivity [47]. Hence, they serve as promising chemotherapeutic agents [43,48].

Researchers have proposed the use of aurilides in combination with ouabain since they potentiate its cytotoxicity [49]. Particularly, these depsipeptides bind to prohibitin 1, a protein located in the mitochondrial inner membrane, which in turn induces the proteolysis of optic atrophy 1, a mitochondrial fusion protein that inhibits cristae remodeling and protects mitochondria from dysfunction [50]. Thus, aurilides induce mitochondrial fragmentation and cell death via apoptosis [51].

#### 2.1.3. Beauvericins

Beauvericins and allobeauvericins, initially isolated from cultures of the fungus *Beauveria bassiana* [52], are a class of cyclo-hexadepsipeptides with core structures consisting of three N-Me-l-phenylalanine units connected alternately with three 2-hydroxy-d-isovaleric acid residues [32]. Interestingly, they can be synthesized in small amounts from bassianolide or beauvericin synthetases [53] or can be assembled as cyclic trimers from three D-Hiv-N-Me-L-amino acid dipeptidol monomers [54].

Beauvericins have shown cancer-targeting effects against lung cancer (A549), epithelial carcinoma (KB), multidrug-resistant cervical cancer (KBv200), and cervix carcinoma (KB-3-1) cells [32]. Moreover, they have demonstrated their effect on tumor cells in vivo (Section 3.2). Beauvericins incorporate themselves into the cell membrane and form a cation-selective channel that disrupts the concentrations of mono- and divalent intracellular cations [55]. They can also increase the intracellular calcium levels and induce apoptosis through oxidative stress [56].

#### 2.1.4. Coibamide A

This marine-derived, cyclic depsipeptide is highly N- and O-methylated [33], and can selectively exert cytotoxicity toward NCI-60 cells, human myeloid cells (HL-60), melanoma cells (LOX-IMVI), glioblastoma cells (SNB-75), and breast cancer cells (MDA-MB-231) [21]. It binds to the Sec61α subunit of the Sec61 protein translocon, non-selectively inhibiting endoplasmic reticulum protein import and subsequently arresting a broad range of membrane-bound and secreted proteins, overall inhibiting cell growth [57].

Coibamide A suppressed the expression of vascular epithelial growth factor A/vascular epithelial growth factor receptor 2 and reduced the tumor size in glioblastoma xenograft models [13]. Furthermore, it potentiated the small-molecule kinase inhibitors, lapatinib and erlotinib, against breast and lung cancers, respectively [58]. Specifically, coibamide A induces mammalian target of rapamycin-independent autophagy via autophagy-related protein 5 and apoptosis through a caspase-dependent pathway [29,59].

Interestingly, since coibamide A is highly N-methylated, it shows metabolic stability, receptor selectivity, and lipophilicity [60]. A loss of N-methylation will disjoin the cyclic and side chain structures or just drastically linearize the molecule [61].

#### 2.1.5. Dehydrodidemnin B

Commonly known as plitidepsin, this cyclodepsipeptide was originally obtained from the Mediterranean tunicate *Aplidium albicans* [34]. Remarkably, dehydrodidemnin B is recognized as a part of the latest generation of didemnins, which exert no toxicity while exhibiting enhanced therapeutic and cancer-targeting effects compared with didemnin B [61]. The latter is a predecessor depsipeptide that caused unsafe side effects like anaphylactic reactions during clinical trials, leading to its suspension from further human research [62].

Multiple studies have evidenced that dehydrodidemnin B causes apoptosis by targeting eukaryotic elongation factor 1A2 [34]. Specifically, it enduringly activates the c-Jun N-terminal kinase, mitogen-activated protein kinase (MAPK), and p38 pathways [63]: three pathways that are involved in stress responses such as inflammation, proliferation, cell death, and survival [64,65,66], subsequently activating Rac1 GTPase and inhibiting phosphatases [67], which eventually results in the release of cytochrome c and apoptosis [63]. Furthermore, dehydrodidemnin B induces cell cycle arrest in a dose-dependent manner [68,69].

Nowadays, dehydrodidemnin B can be artificially synthesized [61], which has elevated it to the status of “drug” in the treatment of different types of cancers in clinical trials (Section 3.6) [70], despite certain reported limitations [71].

#### 2.1.6. Enniatins

These cyclic depsipeptides are produced by strains of fungi such as *Alternaria* spp. and *Verticillium* spp. [35]. Particularly, the mycotoxin enniatin A from *Fusarium* spp., a common contaminant of maize grains, has shown cytotoxicity toward human cervix carcinoma cells (HeLa and KB-3-1) [72,73]. Moreover, enniatins A, B1, and B4 exerted mild cytotoxic effects toward human colon carcinoma cells (HT-29), human epithelial colorectal adenocarcinoma cells (Caco-2), and human liver carcinoma cells (Hep G2) [72].

Enniatin B was shown to halt the growth of cervical cancer cells (Hep 3B and KB-3-1, both human papillomavirus 18-positive and -negative) via apoptosis induction by activating caspase-7, depolarizing the membrane, and cleaving poly (ADP-ribose) polymerase [73]. Enniatin was also reported to cause DNA intercalation, suppress the activity of both topoisomerases I and II, and eventually induce apoptosis through these mechanisms [74].

The lipophilic nature of these compounds allows them to be easily incorporated into the plasma membrane, creating selective cation pores and increasing permeability to create ionic disturbances in the cell [75]. Moreover, enniatin B was reported to be genotoxic to mice [76]. However, genotoxicity was not involved in enniatin B-induced cell death in a hamster lung cell line (V79) [75].

Enniatin B has demonstrated potential cytotoxicity toward human lung cancer cells (VL8), porcine kidney cells (PK-15), embryonic fibroblasts (WI-38), and human glioblastoma (T98-G) cells [75]. In cervical cancer cells, it has shown a synergistic effect with the multi-kinase inhibitor sorafenib, both in vitro and in vivo, by interfering with the extracellular signal-regulated kinase and p38 MAPK pathways [73].

#### 2.1.7. Grassypeptolides

These depsipeptides, especially grassypeptolides F and G, were originally isolated from *Lyngbya* spp., whereas grassypeptolides D and E were derived from *Leptolyngbya* spp. [77]. They constitute a group of 31-membered macrocyclic depsipeptides with high D-amino acid content, one beta-amino acid, and two thiazolines [36].

They can induce cell cycle arrest in the G_1_ phase at low concentrations and in the G_2_/M phase at higher concentrations [13]. Grassypeptolides also display antiproliferative properties by inhibiting the oncogenic transcriptional factor, activator protein-1 [78]. Interestingly, grassypeptolide C demonstrated higher cytotoxicity than grassypeptolide A toward cervical carcinoma (HeLa) and colon adenocarcinoma (HT29) cells, whereas grassypeptolides D and E both displayed significant cytotoxicity toward HeLa and Neuro-2a cells [79].

Grassypeptolides induce apoptosis by inhibiting nuclear factor erythroid 2-related factor 2 [80], which may boost the activity of histone deacetylase inhibitors and promote autophagy-related proteins [81]. Specifically, this pathway is related to survival-induction proteins such as catalase, nicotinamide adenine dinucleotide phosphate dehydrogenase, cyclins A and E, and Bcl-2, among others [82]. Inhibiting these proteins has shown increased cell death, both in vivo and in vitro, demonstrating the importance of this pathway as a possible therapeutic target [81].

#### 2.1.8. Hantupeptins

Three different cytotoxic cyclic depsipeptides, hantupeptins A–C, consisting of a phenyllactic acid and a β-hydroxy acid unit with variable degrees of unsaturation have been isolated from *L. majuscula* [37]. Specifically, they can induce apoptosis in cancer cells [83]. In particular, hantupeptin A, a 19-membered cyclic depsipeptide, has shown potent cytotoxicity against breast cancer (MCF-7) and leukemia cells (MOLT-4) [21], while hantupeptins B and C have exhibited a more modest cytotoxic effect on the same cells [77].

#### 2.1.9. Lagunamides

Belonging to the aurilide class, these depsipeptides have demonstrated cancer-targeting activity as well as antimalarial and antimicrobial effects [21,37]. They consist of four consecutive chiral centers at C37–40 and have an α,β-unsaturated carboxylic acid unit produced by cross-metathesis [38].

Derived from the cyanobacterium *Lyngbya majuscula*, lagunamides exhibit cytotoxicity via mitochondria-mediated apoptosis toward colorectal carcinoma cells (HCT-8), lymphoma cells (P388), lung adenocarcinoma cells (A549), ovarian cancer cells (SK-OV-3), and prostatic adenocarcinoma cells (PC-3) [21,44,84]. Specifically, lagunamides induce apoptosis in these cells by stimulating the production of reactive oxygen species [51].

Additionally, lagunamide A has been shown to induce caspase-mediated mitochondrial apoptosis in lung adenocarcinoma (A549) cells [44]. Likewise, lagunamide D, a macrocyclic depsipeptide derived from *Dichothrix* spp. and *Lyngbya* spp. [85], displayed antiproliferative activity against A549 cells, triggering apoptosis in a time- and dose-dependent manner [86].

#### 2.1.10. Tiahuramides

Tiahuramides, recently described as marine cyclic depsipeptides derived from *L. majuscula* [39], are compounds from the kulolide superfamily consisting of seven amino acids that include a non-proteinogenic residue such as an alkane (tiahuramide C), alkene (tiahuramide B), or alkyne (tiahuramide A) [87]. Interestingly, tiahuramides A–C contain a residue with a triple-, double-, and single-bond fatty acid termination point, respectively [37].

In general, tiahuramides have demonstrated cytotoxicity via both apoptosis and secondary necrosis in animal models compared with necrosis in human neuroblastoma cells (SH-SY5Y) [39]. The half-maximal inhibitory concentrations (IC_50_) of tiahuramides B and C against SH-SY5Y cells were found to be 6.0 μM and 14 μM, respectively [88].

### 2.2. Depsipeptides Inducing Autophagy in Tumor Cells

**Figure 3:**
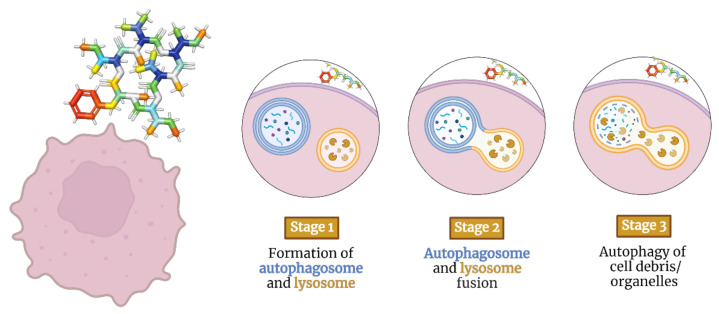
Staging model of depsipeptides inducing autophagy in tumor cells.

Autophagy is the process that uses autophagosomes to deliver cytoplasmic material to the lysosome for degradation [89]. Autophagy-inducing depsipeptides can activate intracellular messengers such as adenosine monophosphate-activated protein kinase and transcription factor EB [90] (Figure 3). They can also inhibit survival-inducing molecules such as hypoxia-inducible factor 1-alpha and mammalian target of rapamycin [29], thereby inducing autophagy through various mechanisms [91,92].

#### Beauvenniatins

Initially isolated from a mycelial extract of *Acremonium* spp., with additional analogs also produced by *Fusarium* and *Isaria* spp. [93], the structure of these peptides is a hybrid between the aromatic and aliphatic cyclodepsipeptides, with moieties of N-Me-valine, N-Me-leucine, and N-Me-phenylalanine (Table 2) [54].

Beauvenniatin F has exhibited strong cytotoxicity against adriamycin-resistant myelogenous leukemia cells (K-562), with an IC_50_ of 2.78 μM, and autophagy-inducing activity at a concentration of 20 μM in green fluorescent protein-light chain 3-stable HeLa cells [94]. These depsipeptides also displayed cytotoxicity against epithelial carcinoma (KB), lymphoma (BC), and lung carcinoma cells (NCI-H187), with IC_50_ values ranging from 1.00 to 2.29 μM as well as against kidney epithelial cells (Vero) at 1.9–5.5 μM [95].

### 2.3. Depsipeptides Inhibiting Elastases

Elastases are a group of proteases that degrade and lyse elastin, one of the main components of the extracellular matrix [96]. These enzymes are also involved in tumor cell migration [97]. Consequently, any dysregulation of elastase may result in the genesis of various diseases such as cancer [98] as well as metastasis [97].

**Figure 4:**
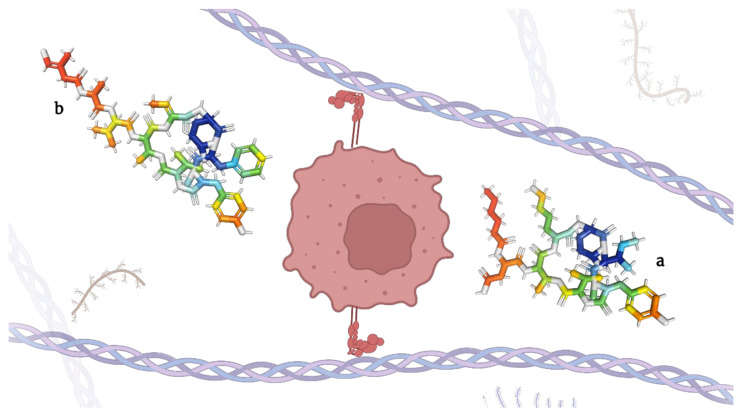
Micropeptins (**a**) and tutuilamides (**b**), depsipeptides capable of inhibiting elastases.

In this section, we elaborate on the depsipeptides with elastase-inhibiting potential (Table 3 and Figure 4).

#### 2.3.1. Micropeptins

Micropeptins are serine protease inhibitors that act against both crustacean and mammalian serine proteases, with proven activity against HeLa cell proteases [101]. They were originally isolated from *Microcystis* spp. from diverse water reservoirs [85] as well as from planktonic fresh water species such as *Anabaena*, *Anabaenopsis*, *Nostoc*, and *Oscillatoria* spp. [102].

Micropeptins are cyclic depsipeptides that possess a 3-amino-6-hydroxy-2-piperidone moiety [99]. Their IC_50_ for elastase inhibition ranges from 4.4 to 50 μM [103]. Interestingly, the elastase inhibitory activity of most micropeptins can be attributed to the leucine moiety in the fifth position from the C-terminus [103].

#### 2.3.2. Tutuilamides A–C

Recently isolated from the cyanobacteria *Schizothrix* spp. (tutuilamides A and B) and *Coleofasciculus* spp. (tutuilamide C) [104], these protease inhibitor peptides have a unique vinyl chloride-containing structure [105]. In tutuilamide A, an additional hydrogen bond between the 4-chloro-3-methylbut-3-enoic acid residue and the backbone amide group of elastase confers a greater inhibitory potency upon this compound [100]. 

Tutuilamides A–C inhibited elastase in lung cancer cells (H-460) with an IC_50_ of 0.53 μM, 1.27 μM, and 4.78 μM, respectively [104].

### 2.4. Depsipeptides Inhibiting Histone Deacetylases

Histone deacetylases constitute a group of enzymes catalyzing the deacetylation of lysine residues found in histones and other proteins [106]. The acetylation of these residues results in a less condensed chromatin structure due to increased space between the nucleosome and DNA, thus modulating cell cycle progression [107]. Interestingly, histone deacetylase inhibitors can induce apoptosis, delay cell cycle progression, and inhibit cancer cell differentiation [30].

**Figure 5:**
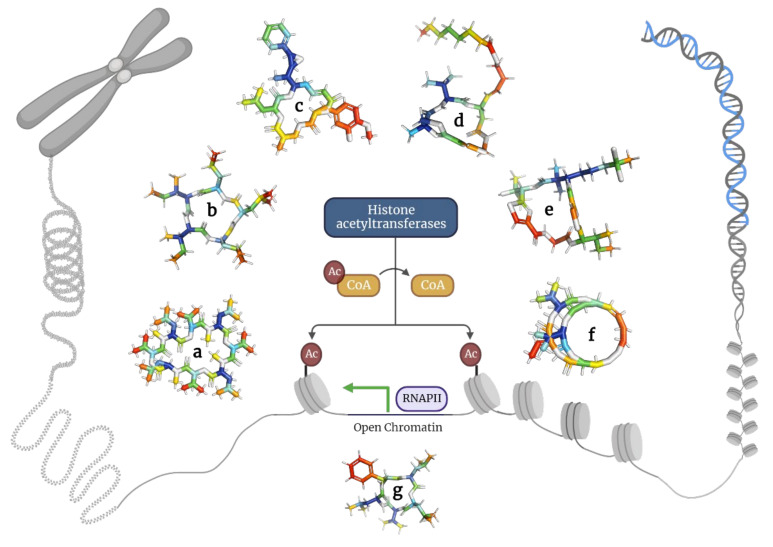
Depsipeptides inhibiting histone acetyltransferases in tumor cells. (**a**) Bassianolide; (**b**) Clavatustides; (**c**) Cryptophycins; (**d**) Largazole; (**e**) Lyngbyabellins; (**f**) Romidepsin; and (**g**) Sansalvamide.

Here, we describe depsipeptides that inhibit histone deacetylases in cancer cells (Figure 5 and Table 4).

#### 2.4.1. Bassianolide

Initially discovered by Suzuki et al. in 1997, bassianolide is a cyclo-oligomer of a tetramer of the depsipeptide D-Hiv-N-Me-L-leucine [108]. Once isolated from *B. bassiana*, *Lecanicillium* spp. (formerly *V. lecanii*), and *Xylaria* spp. BCC1067 [114], this compound was successfully synthesized and demonstrated tumor-targeting activity by inducing G_0_/G_1_ arrest in breast adenocarcinoma cells (MDA-MB-231) [115].

A synthetic analog, (−)bassianolide, is cytotoxic against human lung cancer cells (A549), liver cancer cells (Hep G2), ovarian cancer cells (SK-OV-3), colon adenocarcinoma cells (HCT-15), and MDA-MB-231 cells [108].

#### 2.4.2. Clavatustides A and B

Clavatustides A and B, initially isolated from the cultured mycelia and broth of *Aspergillus clavatus* [13], contain an anthranilic acid dimer and D-phenyl lactic acid [109]. Both depsipeptides suppress the proliferation of hepatocellular carcinoma cell lines (Hep G2, SMMC-7721, and BEL-7402), induce the arrest of Hep G2 cells in the G_1_ phase, and reduce the proportion of cells in the S phase [116].

Cyclin E2 was proved to crucially regulate clavatustide B-induced inhibition of G_1_/S transition in several cancer cell lines [117]. Compared with normal human hepatocytes, Hep G2 cells were more sensitive to clavatustide-induced suppression of proliferation [13]. Clavatustide B also inhibited the growth of human pancreatic epithelioid carcinoma (PANC-1) and prostate cancer (PC-3) cell lines, both of which had shown resistance to chemo- and radiotherapy [118].

#### 2.4.3. Cryptophycin

Isolated from *Nostoc* spp. [119], cryptophycin has demonstrated potent cytotoxicity against HeLa cells [120]. This depsipeptide contains a constrained ring structure that secures its bioactive conformation and protects its bonds from degradation [110]. Specifically, even picomolar concentrations of cryptophycin were found to arrest the cell cycle in the G_2_/M phase [13], interacting with the tubulin interdimer interface, inducing curvature both between and within dimers, inducing changes in α- and β-tubulin [121], and eventually causing depolymerization and apoptosis [43].

Cryptophycin 1 induces apoptosis in ovarian cancer cells (SK-OV-3) by activating caspase-3 [122], also known as CPP32/YAMA/apopain [120]. The cysteine protease CPP32/YAMA/apopain activates apoptosis by targeting specific effector proteins like protein kinase C [123,124]. Moreover, it has inhibited the growth of murine lymphocytic leukemia cells (L1210), epithelial ovarian cancer cells (SK-OV-3), and breast cancer cells (MCF-3), with some studies reporting a cancer-targeting potency nearly 100-fold higher than that of paclitaxel [13].

The effect of this depsipeptide has also been tested in vivo (Section 3.3).

#### 2.4.4. Largazole

Originally isolated from *Symploca* spp. [21], largazole is a strong antiproliferative cyclic depsipeptide that selectively induces cell cycle arrest in human mammary epithelial cells, specifically targeting histone deacetylases [120]. 

Its structure is characterized by a depsipeptide macrocyclic core, with L-valine residues in the C1–C5 portion, an unsaturated lateral chain as a thioester moiety, and a thiazole–thiazoline fragment [111].

Largazole inhibited hypoxia-inducible factor and VEGF receptor 2, showed anti-angiogenic activities in vitro [125], and upregulated p21, a cell cycle inhibitor [126]. It also upregulated E-cadherin and increased its association with γ-catenin in breast cancer cells (MDA-MB-231), thus suppressing their invasiveness [127].

Furthermore, largazole demonstrated potent antiproliferative effects by stimulating histone hyperacetylation in tumor cells, subsequently inducing cell cycle arrest, in cells of colon cancer (HCT116), fibroblastic osteosarcoma (U2OS), glioblastoma multiforme (SF-268 and SF-295), neuroblastoma (IMR-32 and SH-SY5Y), and multiple NCI-60 cell lines [48,128].

Unsurprisingly, largazole has shown important effects on tumor cells when tested in vivo (Section 3.5).

#### 2.4.5. Lyngbyabellins

Lyngbyabellins are secondary metabolites derived from *L. majuscula* and *Moorea bouillonii* [85] with proven antifungal and antibiotic activities [37]. Lyngbyabellins A and B, both of which contain a β-hydroxy acid analog and thiazoline rings [18], have displayed moderate cytotoxicity against human colon cancer (LoVo) and cervical cancer (KB) cells [44]. Both lyngbyabellin A and E possess potent actin polymerization activity [129]. They induce cell cycle arrest in the human Burkitt lymphoma (BL) cell line [48], e.g., lyngbyabellin B arrests BL cells in the G_2_/M phase [130]. Lyngbyabellin E showed significant activity against human lung tumors (NCI-H460) and Neuro-2a cell lines [131], and lyngbyabellin N against colorectal carcinoma cells (HCT116) [44].

#### 2.4.6. Romidepsin

Romidepsin is a potent, structurally unique, selective histone deacetylase inhibitor approved by the Food and Drug Administration to treat both cutaneous and peripheral T-cell lymphoma [132]. It is a cage-shaped, bicyclic pentapeptide composed of D-valine, D-cysteine, dehydrobutyrine, L-valine, and 3-hydroxy-7-mercapto-4-heptenoic acid [112].

Romidepsin increases both the mRNA and protein levels of p53, p21, caspases 3, 7, and 8, and poly (ADP-ribose) polymerases, thus inducing apoptosis and inhibiting the proliferation of endometrial cancer cells [133]. It synergistically acts with a wide array of chemotherapeutics such as lenalidomide for T-cell lymphomas or rituximab for B-cell lymphomas [132].

#### 2.4.7. Sansalvamide A

This cyclic depsipeptide, isolated from the marine fungus *Fusarium* spp. [134], exhibits broad antitumor activity against 60 cancer cell lines including those of breast (MDA-MB-231), colon (COLO 205 and HCT116), melanoma (WM-155 and SK-MEL-2), pancreatic, (AsPC-1), and prostate cancers (PC-3) [21,113,135,136]. Its chemical structure comprises four proteogenic amino acids and one hydroxyl acid [113]. Specifically, sansalvamide A demonstrated higher potency against colorectal carcinoma (COLO 205) and melanoma cells (SK-MEL-2) [43].

Sansalvamide A arrests cells in the G_1_ phase, inhibits topoisomerase I [21,135], and subsequently triggers apoptosis of tumor cells [48]. It causes in vitro pancreatic cancer cell death by downregulating cyclins A, CD4, D1, E, and upregulating p21, resulting in cell cycle arrest in the G_0_/G_1_ phase [13,135]. N-methylation of sansalvamide A enhances its antitumor cytotoxicity and selectivity [137]. Its derivative H-10 has also shown antiproliferative effects, particularly against murine melanoma cells (B16), by inducing apoptosis [138].

### 2.5. Depsipeptides Disrupting Microfilaments

Microtubule–actin interactions are fundamental for many cellular processes such as cell motility, cell division, and cytoskeleton dynamics [139]. Many depsipeptides derived from bacteria exert activities against microfilaments, causing complete depolymerization of microtubules and hyper-polymerization of actin [140]. Moreover, they have shown the ability to destabilize microtubules, inducing caspase and Bcl-2 activation, eventually resulting in cell cycle arrest and apoptosis [141].

**Figure 6:**
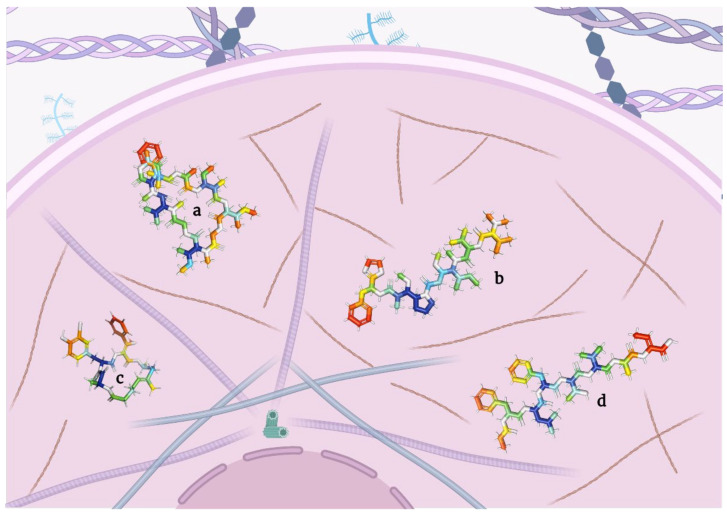
Models of depsipeptides inducing microfilament disruption in tumor cells. (**a**) Desmethoxymajusculamide C; (**b**) Dolastatin 10; (**c**) Miuraenamide; (**d**) Nobilamide I.

In this subsection, we explore the depsipeptides that disrupt microfilaments in tumor cells (Figure 6 and Table 5).

#### 2.5.1. Desmethoxymajusculamide C

Derived also from *L. majuscula* [146], this cyclic depsipeptide has demonstrated selective and potent cancer-targeting activity against human colorectal (HCT116) and breast (MDA-MB-435, MDA-MB-231, and MCF7) cancer cells by disrupting cellular microfilament networks [43], specifically via the depolymerization of the actin cytoskeleton [44].

Notwithstanding, desmethoxymajusculamide C displays cancer-targeting activities both in its cyclic and ring-opened forms [142]. Both the linear and the cyclic forms of this depsipeptide caused the same cytotoxicity [13].

#### 2.5.2. Dolastatin 10

Dolastatin 10, originally isolated in the 1980s from the mollusk *D. auricularia* in the Indian Ocean, is a pentapeptide that contains three unique amino acid residues [147] and five subunits: L-valine, N,N-dimethyl valine, (3*R*,4*S*,5*S*)-dolaisuleucine, (2*R*,3*R*,4*S*)-dolaproine, and a protected (*S*)-dolaphenine [143,148].

This depsipeptide induces apoptosis by interacting with microtubulin and interfering with microtubule assembly [149]. Since dolastatin 10 was first discovered, its cytotoxic activity was deemed more potent than that of anticancer drugs, and due to its simple chemical structure, it was considered a promising anticancer agent [147].

Consequently, this depsipeptide, along with some of its derivate analogs such as soblidotin and tasidotin has reached clinical trials with significant results (Section 3.7 and Section 3.8).

#### 2.5.3. Miuraenamide

Derived from the myxobacterium *Paraliomyxa miuraensis* [150], miuraenamides are cyclic depsipeptides containing a polyketide unit and an N-terminal alanine bound to an N-methylated halogenated aromatic amino acid, specifically tyrosine [144]. They inhibit cell migration, favor actin polymerization by stabilizing the oligomers formed during nucleation, and promote the assembly and stabilization of such filaments [151].

Moreover, treatment with miuraenamide impairs the migration and enhances the nuclear stiffness of ovarian epithelial carcinoma cells (SK-OV-3) cells. It downregulates the expression of proteins in the Wnt pathway and myocardin-related transcription factor-associated proteins [152]. Additionally, miuraenamide A has shown the ability to significantly change the morphology of the cytoplasm and nucleus of HeLa cells [153].

#### 2.5.4. Nobilamide I

Nobilamide was originally isolated from a mollusk-associated bacterium, *Streptomyces* sp. CN48 [154]. It is a linear heptapeptide with a Z-di-dehydroamino butanoic acid moiety, which is considered to be responsible for its inhibitory effects on the transient receptor potential vanilloid-1 cation channel [145].

Nobilamide I inhibits cancer cell motility and tumorigenesis in gastric (AGS), lung (A549), and colon carcinoma (Caco-2) cells by suppressing the expression of E- and N-cadherins, the transcription factors Snail, Slug, and Twist, and matrix metalloproteinase 2/9 [155].

### 2.6. Depsipeptides Inhibiting Cell Growth

The loss of normal cell cycle regulation is one of the cornerstones of human cancer emergence [156]. Thus, compounds that inhibit or terminate mitosis have been the emphasis of clinical trials for many years, with some achieving significant success [157]. Certain depsipeptides are of particular interest in this field [120,122], mainly skyllamycins and stereocalpin compounds that inhibit transcription factors and survival signaling [158,159].

**Figure 7:**
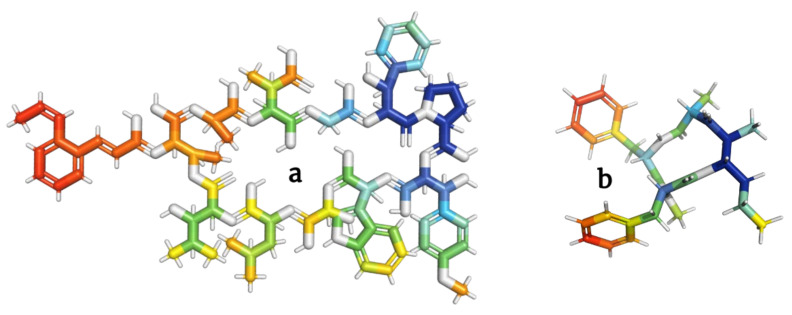
Models of skyllamycins (**a**) and stereocalpin (**b**) depsipeptides inhibiting the growth of tumor cells.

Thus, we delved into the depsipeptides that inhibit cell growth (Table 6 and Figure 7).

#### 2.6.1. Skyllamycins

Skyllamycins are a group of non-ribosomal cyclic depsipeptides produced by *Streptomyces* spp. [162], constituted, among others, by D-leucine, D-tryptophan, a pseudo-N-terminal cinnamic acid residue, and a β-Me-aspartic acid residue [160]. Additionally, these compounds possess a particularly rare α-OH-glycine residue [163] and a unique cinnamoyl side chain connected to a serine/threonine residue [162] (Figure 7).

Skyllamycin A inhibits the platelet-derived growth factor pathway [158], resulting in the inhibition of mitosis in tumor cells [164]. Since it selectively blocks the binding of the platelet-derived growth factor BB homodimer to the β-receptor, skyllamycin A could exert effects like those of imatinib on cancer, particularly against chronic myelogenous leukemia [165].

#### 2.6.2. Stereocalpin A

Initially isolated from the Antarctic lichen *Stereocaulon alpinum*, stereocalpin A is a cyclic depsipeptide with a unique structure involving a 5-hydroxy-2,4-dimethyl-3-oxo-octanoic acid [161]. It has demonstrated moderate cytotoxicity against three solid tumor cell lines: B16-F10, Hep G2, and HT-29 [166].

Stereocalpin A inhibited tumorigenesis by preventing the accumulation of tumor necrosis factor-α-induced adhesion molecules by inhibiting MAPK, protein kinase B (Akt), and the pro-inflammatory transcription factor nuclear factor-κB [159]. This mechanism also suggests that this depsipeptide can exert a protective effect by modulating inflammation within atherosclerotic lesions [166].

### 2.7. Depsipeptides Inhibiting Topoisomerases

Topoisomerases are enzymes that relieve torsional stress within DNA during replication and transcription by cleaving one strand to unwind the supercoiled DNA [167,168]. Inhibiting these enzymes leads to DNA damage, nicotinamide adenine dinucleotide phosphate oxidase-dependent generation of reactive oxygen species, p21 activation, and eventual cell cycle arrest and senescence [169].

**Figure 8:**
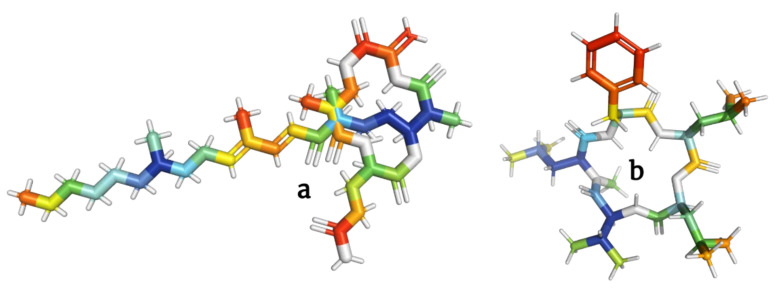
Models of fusaristatin A (**a**) and neo-N-methylsansalvamide (**b**) depsipeptides targeting topoisomerases in tumor cells.

Here, we describe the relevant depsipeptides targeting topoisomerases in cancer cells (Table 7 and Figure 8).

#### 2.7.1. Fusaristatin A

Fusaristatins A and B, isolated from the endophytic fungus *Fusarium* spp. [171], contain β-aminoisobutyric acid, dehydroalanine, and glutamine moieties [170]. Both compounds moderately inhibited topoisomerases I and II as well as the growth of lung cancer (LU65) cells [172]. Moreover, they exhibited cytotoxicity against non-small-cell lung cancer (SCLC, NCI-H460) and melanoma cells (MDA-MB-435) with an IC_50_ of 8.15 μM [173,174].

#### 2.7.2. N-Methylsansalvamide

N-methylsansalvamide, an analog of sansalvamide A, is a cytotoxic cyclic pentadepsipeptide derived from the marine fungus *Fusarium solani* KCCM90040 [175,176]. In vitro, it inhibited the growth of human lung cancer cells (A549), ovarian cancer cells (SK-OV-3), melanoma (SK-MEL-2), and uterine sarcoma (MES-SA) cells with IC_50_ values ranging from 10 to 14.74 μM [13]. Specifically, this novel depsipeptide inhibited topoisomerase I and inhibited the growth of colorectal carcinoma (HCT-15) cells [28].

Interestingly, neo-N-methylsansalvamide effectively reversed multidrug resistance in tumor cells and may be used as a resistance reversal agent in chemotherapeutic regimens [28].

### 2.8. Oncosis

Oncosis is a form of programmed cell death characterized by organelle swelling and membrane disruption [177]. It is a type of cell injury that involves energy depletion, impairment of ionic pumps, swelling, dilation of the Golgi apparatus and endoplasmic reticulum, chromatin clumping, and the formation of cytoplasmic blebs [178].

**Figure 9:**
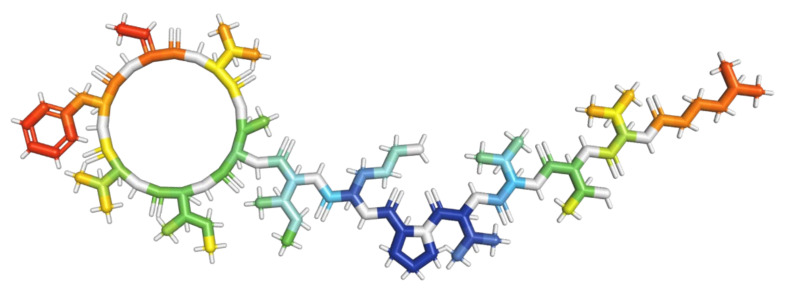
Model of kahalalide F: the only depsipeptide inducing oncosis in tumor cells.

Compared with apoptosis, oncosis eventually results in necrosis with karyolysis and cell swelling, instead of karyorrhexis and cell shrinkage [179]. One of the most studied oncosis-inducing depsipeptides in cancer cells is kahalalide F (Figure 9 and Table 8), a molecule that causes disruption of the mitochondrial membrane potential and alters the permeability of lysosomal membranes [91].

#### Kahalalide

Kahalalide F is a tridecapeptide with a C75 cyclic structure containing dehydro-aminobutyric acid and a 19-membered ring formed by five residues [18]. It was originally isolated from the sea slug *Elysia rufescens* and has shown that it can induce cancer cell necrosis [180].

Kahalalide F has displayed potent cytotoxicity against cancer cells of the breast (BT-474, MCF7, MDA-231, and SK-BR-3), colon (HT29 and LoVo), liver (Hep G2), lung adenocarcinoma (A549), ovaries, and prostate (DU145 and LNCaP) [181,182,183]. It targets lysosomes and induces extreme vacuolization and swelling [18,180].

Specifically, it induces karyolysis and necrosis or oncosis, with IC_50_ values between 0.2 and 10 μM [184] via aggregation of DNA, deactivation of Akt and receptor tyrosine kinase (ErbB/Her), eventually creating pores in the cell membrane [181]. Recently, modifying kahalalide F by coupling with a biotinylated linker, prepared from biotin and tetra-ethylene glycol, has enhanced its cancer-targeting properties via the human ribosomal protein S25 [185].

## 3. Depsipeptides Studied In Vivo and Clinical Trials

While in vitro testing is the first step to assess candidates in the drug development pathway, in vivo studies represent the second stage, in which molecules reveal both their potential and limitations in physiological conditions [186]. Clinical trials represent the third stage of this effort, intending to detect variabilities derived from a matrix effect in the human body [19].

The mechanism of action of depsipeptides in vivo relies on apoptotic, necrotic, or lytic phenomena [20]. Unfortunately, in vivo studies demonstrating the potent inhibition of different cancers are limited, mainly due to toxicity, which hinders the cancer-targeting activity of these peptides and precludes them from reaching clinical trials [44]. Regardless, some depsipeptides have achieved relevant milestones in cancer therapy, based on their clinical trial reports [187].

We have delved into such depsipeptides here, which have displayed tumor-targeting capabilities in vivo and during clinical trials.

### 3.1. Apratoxin

Apratoxin can downregulate receptor tyrosine kinases and VEGF-A in colorectal tumor models [188]. Specifically, apratoxin S4 demonstrated potent anti-angiogenic activity as it prevented pathological ocular neovascularization in vivo [126].

Nonetheless, apratoxin was found to be ineffective against breast tumor cells [189]. Furthermore, exposure to apratoxin A caused pancreatic atrophy and significant toxicity, thus limiting its widespread use [190].

### 3.2. Beauvericin

The in vivo cancer-targeting activity of beauvericin has been assessed in an allograft and xenograft mouse model [32]. After three days of a subcutaneous injection of murine CT26 colon carcinoma cells in BALB/c mice, 5 mg/kg/day of beauvericin treatment markedly reduced tumor growth after a second treatment cycle two weeks later [32,191].

The efficacy of beauvericin was also demonstrated in a cervix carcinoma xenograft model (KB-3-1) where it significantly reduced tumor volumes [191]. The drug accumulated in tumor tissues and necrotic areas within tumors significantly increased [56].

### 3.3. Cryptophycins

These cyclic depsipeptides of a bacterial origin and exerting significant cytotoxicity against various cancer cell lines including multidrug-resistant tumors [192], demonstrated improved biological activity in vivo compared with paclitaxel and vinblastine, with a 100- to 1000-fold greater activity against the same cell lines [193].

A novel compound composed of acetazolamide and cryptophycin reduced in vivo tumor growth in nude mice bearing renal cell carcinoma cells (SK-RC-52) [192]. Newer compounds like cryptophycin-8 have demonstrated lower toxicity and greater therapeutic efficacy than cryptophycin-1 in vivo [194].

### 3.4. Dolastatin 10

Aside from its proven antiproliferative activity [149], dolastatin 10 showed in vivo anticancer activity against multidrug-resistant diffuse large cell lymphoma (WSU-DLCL2) [195], SCLC (NCI-H69, -H82, -H446, -H510) [196], murine leukemia (P388 and L1210), melanoma (B16 and LOX), sarcoma (M5076), and breast cancer (MX-1) cell lines in xenograft murine models [184].

Dolastatin 10 was evaluated in SCID mice with metastatic and subcutaneous SCLC xenoplantation models, where it demonstrated proapoptotic abilities at an IC_50_ in the range of 0.032–0.184 nM [147]. Moreover, it showed potent antiproliferative activity against murine leukemia cells, with an effective dose of 50 of 4.6 × 10^−5^ mcg/mL [197].

Recently, dolastatin 10 started phase II clinical trials in patients with non-SCLC, metastatic melanoma, colon, breast, and multidrug-resistant ovarian cancer, with demonstrated toxicity against these tumors [149]. Interestingly, dolastatin 10 showed more potent activity than paclitaxel or vinblastine in a variety of murine cancer models [198].

### 3.5. Largazole

This macrocyclic depsipeptide, containing a thiazole unit linked to a 4-Me-thiazoline, a non-modified L-valine residue, and a thioester responsible for its mechanism of action [199,200], has exhibited a wide range of in vitro and in vivo biological activities such as antitumor, anti-osteogenic, and antifibrotic [201]. In particular, largazole potently stimulates histone hyperacetylation by inducing apoptosis in murine colon cancer (HCT116) cells [200]. An intraperitoneal dose of 5 mg/kg of a largazole peptide isostere effectively inhibited tumor growth in a non-SCLC (A549) xenograft model [202].

### 3.6. Plitidepsin (Dehydrodidemnin B)

Initially, plitidepsin potently inhibited osteoclast differentiation and bone resorptive activity in vivo [203]. Moreover, it could halt the progression of the myc-oncogenic signaling pathway by inhibiting protein synthesis and enzymatic activity, thereby demonstrating potent efficacy in phase I trials, especially against medullary thyroid carcinoma [204].

Subsequently, plitidepsin entered various phase II and III trials for the treatment of multiple cancers [205], where it displayed cancer-targeting effects against BL, diffuse large B-cell lymphoma (HT and RL), leukemia (MOLT-4), multiple myeloma (5TMM), pancreatic cancer, and T-cell lymphoma [206,207]. These clinical trials further underscore the promise of plitidepsin as a cancer-targeting agent [68,208,209].

Since 2018, after completing a phase III trial, plitidepsin is currently approved as a therapeutic agent for relapsed/refractory multiple myeloma patients [210]. Interestingly, studies are testing its combination with gemcitabine, sorafenib [211], and dexamethasone [70].

Currently, plitidepsin is under further clinical trials [70]. The marketed drug is being tested against solid and hematological malignancies such as T-cell lymphoma, leukemia, and prostate cancer [212]. These phase II trials, which involve the intravenous application of 5 mg/m^2^ of plitidepsin, have yielded heterogeneous results: some patients achieved disease stabilization, whereas only a few achieved a partial response [211].

### 3.7. Soblidotin

Soblidotin, an analog of dolastatin 10 with tubulin-inhibiting and vascular-disrupting abilities [18], has demonstrated in vivo anticancer activity against human xenograft models of breast (MX-1) and lung (LX-1) cancer [213]. Injected intravenously in mice, it significantly inhibited the growth of leukemia cells (P388) and reduced the tumor volume of three solid tumor cell lines: colon-26 adenocarcinoma, B16 melanoma, and M5076 sarcoma [197].

Soblidotin demonstrated in vivo antivascular effects against tumors overexpressing VEGF in nude BALB/c and CDF1 mice inoculated with SCLC (SBC-3/VEGF) [214] and showed antitumor activity against tumors resistant to docetaxel, paclitaxel, and vincristine [149]. In 2002, soblidotin began phase I clinical trials in Europe, Japan, and the USA, and is currently in phases II and III with multiple companies [18].

Moreover, soblidotin was demonstrated to be superior or at least comparable to paclitaxel and vincristine in their antimicrotubular activities [198]. However, reports of significant hematological toxicity have stalled the progression of soblidotin as a potential anticancer agent [215].

### 3.8. Tasidotin

Tasidotin is a third-generation analog of dolastatin 15 that is metabolically stable due to its resistance to hydrolysis [149]. It showed in vivo anticancer activity in preclinical models of pediatric sarcomas [216], LOX melanoma xenografts [217], and xenograft models of breast, ovarian, prostate, and colon cancer [216].

Additionally, tasidotin has also been effective in vivo against a murine P388 leukemia model [216]. Tasidotin induces cell cycle arrest in the G_2_ and M phases and also inhibits tubulin polymerization, even at low concentrations, demonstrating activity against rhabdomyosarcoma, synovial sarcoma, and osteosarcoma [149].

## 4. Current Methods for Purification and Synthesis of Depsipeptides

### 4.1. Proved Methods for Purifying Depsipeptides

Nowadays, depsipeptides can be purified using different methods [218,219]. When derived from natural sources, the organism-produced depsipeptides can be analyzed using high-performance liquid chromatography (HPLC) [220,221]. Some studies have coupled a photo-diode array and used ultra-performance liquid chromatography in tandem with high-resolution mass spectrometry [222].

Frequently, in vitro and even in vivo assays are performed simultaneously during purification to analyze the biological properties of interest [223]. Yu et al. used reversed-phase HPLC (RP-HPLC) to purify and quantify depsipeptides and short oligomers of glycine [220]. In 2006, Matsuo et al. also used RP-HPLC to purify urukthapelstatin [224], a depsipeptide with inhibitory effects on human lung cancer (A549) cells [221].

RP-HPLC was also used to purify trikoveramides A–C, peptides that belong to the kulolide superfamily and inhibited the growth of human leukemia cells (MOLT-4) [225]. The well-known anticancer peptide, coibamide A, was also purified by RP-HPLC [226]. In 2015, Kaur et al. achieved the purification of the novel cyclic depsipeptide, YM-280193, with the ability to inhibit platelet aggregation [227].

### 4.2. Synthesis of Depsipeptides

Depsipeptides were originally synthesized to improve the solubility of ultra-long peptides by adding an ester-linked residue that would alter the linear structure [228]. In addition to the presence of at least one ester and one amide bond in their structures, most depsipeptides contain a wide variety of acyl groups and moieties added through the assembly line [16].

Certain authors have proposed the synthesis of these peptides using solid-phase peptide synthesis with a fluorenylmethoxycarbonyl (Fmoc)-protected N-terminus, lysine, or aspartic acid as the charged entities, and lactic acid as the ester moiety to maintain hydrophobicity [219]. A stepwise Fmoc-based solid-phase methodology to create a highly complex depsipeptide has been reported, with the addition of small amounts of organic acids to improve this stepwise approach and minimize secondary reactions [229].

For instance, coibamide A has been synthesized using Fmoc-based solid-phase synthesis, but with the initial step being the attachment of the Fmoc-Tyr-allyl ester to the free hydroxyl group on a 2-chlorotrityl chloride resin [226]. Another depsipeptide, teixobactin, and its analogs have also been synthesized using Fmoc-based solid-phase synthesis [230].

A recent modification of the Fmoc-based solid-phase synthesis involved the use of ultrasonication methods, an intervention that saved both material and reaction time while improving the synthesis yield of complex peptide sequences [231]. A novel and efficient approach to generate depsipeptides emerged in the form of isocyanide-based consecutive Bargellini–Passerini multicomponent reactions [232].

## 5. Recognized Disadvantages of Depsipeptides

Even though depsipeptides are derived from natural resources, their commercialization requires synthetic production, which generates a vast amount of waste [233]. To tackle these issues, cyclization has been proposed to minimize dimerization and oligomerization, which in turn involves high dilutions of the peptide [234]. However, a synthetic route needs to be designed, optimized, and tailored to each particular depsipeptide, rendering a general synthetic method impossible [229].

Synthetic depsipeptides have to replicate the capability of modulating DNA methylation, acetylation, and histone methylation through specific biomolecules with unique structural characteristics [235], making this a complex, strenuous, and most likely, high-cost process. Furthermore, these peptides tend to be ionophoric in nature and require metal salt templates to be added to the macrocyclization reactions as they enhance the rate of cyclization [233]. Moreover, the cell-binding capacity of these peptides and the uncertain toxicity of their degradation products are some other concerns [236].

## 6. Discussion

Depsipeptides are molecules with remarkable features that can be exploited for both health and nutrition. Currently, researchers are using these molecules in innovative ways (e.g., as radio-diagnostic imaging agents) [237]. Their cancer-targeting capability (“anticancer”), which we have addressed in this review, has been studied for numerous years.

As depicted in the different sections of this review, cyclic depsipeptides are quite common [92]. Several authors sort them based on their structure, mainly according to the existence and number of ester bonds, if these occur regularly or irregularly, and the position of ester oxygen (i.e., α or β) [238,239]. However, an amendment to this system has been proposed, which classifies depsipeptides according to the introduction of the ester bond as well as the origin of the ester hydroxy group, among other revisions [16].

This latter modification proved to be important for defining the structure of depsipeptides in future research projects. We observed that the number of depsipeptides having cytotoxic effects on cancer cells decreases as the number of ester groups increases. Likewise, the number of candidate depsipeptides dramatically decreased in different sources as the number of ester groups increased.

To date, studies on depsipeptides have been primarily focused on fungi, insects, and marine lifeforms. No depsipeptides have been reported from common food sources like maize or wheat, both of which are widely consumed worldwide [240]. This is attributed to the synthesis of these compounds, which requires the participation of multi-domain non-ribosomal peptide synthetases. These are a part of the large biochemical arsenal encoded in microbial genomes [16,241].

Likewise, depsipeptides, as secondary metabolites, are especially copious in filamentous fungi than in plants [242]. Cutting-edge peptides are being engineered from proteins of these organisms with relevant activities for cancer therapy [8,243]. Furthermore, proposals for the use of other common substrates to produce bioactive peptides such as fruits and vegetables have been proposed, but their low level of protein (0.5–3.9%) limits their use in this setting [11].

Hence, we encourage specialists to delve into the native sequences of large-scale consumed foods, intending to find candidate peptides prone to being cyclized and enhanced as needed, but always according to in silico prediction and complementary design such as in linear peptides [244]. The cyclic nature of depsipeptides is reported to bestow upon them advantages such as increasing both protease resistance and protein-binding capability [16].

Most of the molecules we covered in this review eventually induce apoptosis. Certain molecules such as micropeptins and the tutuilamides A–C have demonstrated elastase inhibition properties [103,245], making them suitable anticancer agents because the levels of this protease are significantly elevated in various cancers [246]. Likewise, some specific depsipeptides that induce autophagy include apratoxin A, coibamide A, and plitidepsin, which induces macroautophagy via the autophagy-related protein 5–ubiquitin system [29,247].

Although this review encompasses notable findings of specific depsipeptides and generally known disadvantages, certain drawbacks of specific candidates must be highlighted. For instance, apratoxin has been shown to be ineffective against breast tumor cells [189]. Moreover, exposure to it caused pancreatic atrophy and significant toxicity in vivo, which limited its widespread use [190].

Likewise, dolastatin 15, a compound quite similar to dolastatin 10 (addressed in Section 2.5.2 and Section 3.4 herein), demonstrated an effect on growth and differentiation in leukemia cell lines and G_2_/M cell cycle arrest in human myeloma cell lines during in vivo trials [149]. However, further studies revealed that it was nearly seven times less active than its counterpart [140]. This slightly weaker in vivo activity was also reported for other molecules such as dolastatin 10 and isodolastatin H [248].

Based on our analysis, the only depsipeptide that might be considered for encapsulation studies on food matrices would be dehydrodidemnin B (plitidepsin). Thus far, two nanoparticles formulated with plitidepsin have produced successful results when targeting renal cancer in a xenograft model [216]. Notably, these nanoparticles achieved a similar targeting result as Cremophor, a marketed adjuvant for hydrophobic and insoluble drugs for cancer treatment [181].

Nonetheless, assessing the effect of the route of administration of any candidate depsipeptide would be mandatory for food enrichment purposes, principally because these molecules lack specific properties to be encapsulated within lipid vesicles, for instance, some contain arginine residues within their amino acid sequence [249]. Some synthetic depsipeptides derived from human C-reactive protein, however, have been successfully encapsulated in multi-lamellar vesicles [250].

Even with promising in vitro results for multiple cyclic peptides, most of the studies delving into the toxicity profile of these molecules are significantly lacking in the effects on normal cells [10,193]. Other authors have generalized the applicability of a few metabolites that have progressed in early phases of clinical trials [120], claims that may prove deleterious for these molecules’ approval and use.

Likewise, emphasis should be given to the characteristics of many review studies on depsipeptides, most of which delve into significant detail regarding promising findings [120,181,251], but fail to discuss the reliability of results and few refer to quality control measures and the reproducibility of results. Nonetheless, other authors have excelled in describing their methods, strategies, and limitations [252], thus yielding important information that will most likely pave the way for the rapid development of novel and reliable depsipeptides.

Therefore, we encourage researchers to reevaluate the concept of using these molecules as food additives to achieve an “anticancer” effect from the consumption of a routine diet. Studies should be directed toward effective encapsulation techniques and the potential tumor-targeting “pharmacokinetics” of these elite candidates.

## 7. Conclusions

Depsipeptides are complex molecules that have attracted significant interest in the field of cancer therapeutics. To date, several elite cancer-targeting candidates have been identified, and investigations range from in vitro assays to clinical trials. This could indeed be the key for functional food purposes.

However, in their pristine form, depsipeptides are still far from being introduced into foods, mainly due to their structure and susceptibility to the matrix environment. Purification and synthesis methods have certainly enhanced the proficiency of depsipeptide management.

Consequently, research efforts should focus on effective encapsulation techniques while retaining their cancer-targeting capacity and considering the physiological impact of their degradation products, so that these elite candidates can be used as cancer therapeutics in the future.

## Figures and Tables

**Table 1:** Formula and molecular weight of depsipeptides inducing apoptosis in tumor cells.

Key	Depsipeptide	IUPAC Condensed Formula	Molecular Weight (g/mol)	Ref.
a	Apratoxin	Cyclo[N(Me)Ala-N(Me)Ile-Pro-Unk-Tyr(Me)]	840.1	[31]
b	Aurilide	Cyclo[N(Me)Ala-Unk-Val-D-N(Me)Leu-Sar-Val]	834.1	[21]
c	Beauvericin	Cyclo[D-Oval-N(Me)Phe-D-Oval-N(Me)Phe-D-Oval-N(Me)Phe]	783.9	[32]
d	Coibamide	N(Me2)Val-Oval-N(Me)Ser(Me)-N(Me)Leu-N(Me)Thr(1)-N(Me)Ser(Me)-N(Me)Ile-Ala-N(Me)Leu-Tyr(Me)-N(Me)Ala-(1)	1287.6	[33]
e	Dehydrodidemnin	Pyruvoyl-Pro-D-N(Me)Leu-D-Thr(1)-Unk-Leu-Pro-DL-N(Me)Tyr(Me)-(1)	1110.3	[34]
f	Enniatin	Cyclo[DL-Oval-DL-N(Me)xiIle-DL-Oval-DL-N(Me)xiIle-DL-Oval-DL-N(Me)xiIle]	681.9	[35]
g	Grassypeptolide	Cyclo[D-N(Me)Leu-D-aThr-Unk-N(Me)Val-Pro-Unk]	1102.4	[36]
h	Hantupeptin	Cyclo[N(Me)Ile-Ophe-Pro-N(Me)Val-Unk-Val]	740.9	[37]
i	Lagunamide	Cyclo[Ala-D-N(Me)Phe-Sar-aIle-N(Me)Ala-Unk]	842.1	[38]
j	Tiahuramide	Cyclo[N(Me)Ile-Unk-Val-N(Me)Val-Ophe-Pro]	736.9	[39]

**Table 2:** Formula and molecular weight of beauvenniatin, a depsipeptide triggering autophagy in tumor cells.

Depsipeptide	IUPAC Condensed Formula	Molecular Weight (g/mol)	Ref.
Beauvenniatin	Cyclo[D-OaIle-N(Me)Phe-D-OaIle-N(Me)Val-D-OaIle-N(Me)Val]	729.9	[54]

**Table 3:** Formula and molecular weight of depsipeptides inhibiting elastases in tumor cells.

Key	Depsipeptide	IUPAC Formula	Molecular Weight (g/mol)	Ref.
a	Micropeptins	(3*S*)-4-[[(2*S*,5*S*,8*S*,11*R*,12*S*,15*S*,18*S*,21*R*)-15-(4-aminobutyl)-2-[(2*S*)-butan-2-yl]-21-hydroxy-5-[(4-hydroxyphenyl)methyl]-4,11-dimethyl-3,6,9,13,16,22-hexaoxo-8-propan-2-yl-10-oxa-1,4,7,14,17-pentazabiCyclo [16.3.1]docosan-12-yl]amino]-3-(hexanoylamino)-4-oxobutanoic acid	945.1	[99]
b	Tutuilamides	(2*S*)-*N*-[(2*S*,5*S*,8*S*,11*R*,12*S*,15*Z*,18*S*,21*R*)-2-benzyl-15-ethylidene-21-hydroxy-5-[(4-hydroxyphenyl)methyl]-4,11-dimethyl-3,6,9,13,16,22-hexaoxo-8-propan-2-yl-10-oxa-1,4,7,14,17-pentazabiCyclo [16.3.1]docosan-12-yl]-2-[[(2*S*)-2-[[I-4-chloro-3-methylbut-3-enoyl]amino]propanoyl]amino]-3-methylbutanamide	1007.6	[100]

**Table 4:** Formula and molecular weight of depsipeptides inhibiting histone deacetylases in tumor cells.

Key	Depsipeptide	IUPAC Formula	Molecular Weight (g/mol)	Ref.
a	Bassianolide	Cyclo[N(Me)Leu-D-Oval-N(Me)Leu-D-Oval-N(Me)Leu-D-Oval-N(Me)Leu-D-Oval]	909.2	[108]
b	Clavatustide	Cyclo [2Abz-2Abz-D-Ophe-N(Et)Gly]	471.5	[109]
c	Cryptophycin	10-[(3-chloro-4-methoxyphenyl)methyl]-6-methyl-3-(2-methylpropyl)-16-[1-(3-phenyloxiran-2-yl)ethyl]-1,4-dioxa-8,11-diazacyclohexadec-13-ene-2,5,9,12-tetrone	655.2	[110]
d	Largazole	S-[I-4-[(5*R*,8*S*,11*S*)-5-Me-6,9,13-trioxo-8-propan-2-yl-10-oxa-3,17-dithia-7,14,19,20-tetrazatriCyclo [14.2.1.12,5]icosa-1(18),2(20),16(19)-trien-11-yl]but-3-enyl] octanethioate	622.9	[111]
e	Lyngbyabellin	(7*S*,14*S*,18*S*)-7-[(2*S*)-butan-2-yl]-14-(4,4-dichloropentyl)-18-(2-hydroxypropan-2-yl)-15,15-dimethyl-13,17-dioxa-9,20-dithia-3,6,22,23-tetrazatriCyclo [17.2.1.18,11]tricosa-1(21),8(23),10,19(22)-tetraene-2,5,12,16-tetrone	691.7	[18]
f	Romidepsin	7-ethylidene-4,21-di(propan-2-yl)-2-oxa-12,13-dithia-5,8,20,23-tetrazabiCyclo [8.7.6]tricos-16-ene-3,6,9,19,22-pentone	540.7	[112]
g	Sansalvamide	Cyclo[L-leucyl-N-oxa-L-leucyl-L-valyl-L-leucyl-L-phenylalanyl]	586.8	[113]

**Table 5:** Formula and molecular weight of depsipeptides inducing microfilament disruption in tumor cells.

Key	Depsipeptide	IUPAC Formula	Molecular Weight (g/mol)	Ref.
a	Desmethoxymajusculamide C	Cyclo[Ala-Unk-Ala-Unk-Gly-N(Me)Ile-Gly-N(Me)Val-N(Me)Phe]	955.2	[142]
b	Dolastatin	N(Me2)Val-Val-Unk	785.1	[143]
c	Miuraenamide	(3*E*,15*E*)-6-[(3-bromo-4-hydroxyphenyl)methyl]-3-[methoxy(phenyl)methylidene]-7,9,16,19-tetramethyl-1-oxa-4,7,10-triazacyclononadec-15-ene-2,5,8,11-tetrone	684.6	[144]
d	Nobilamide	Propionyl-D-Phe-D-Leu-Phe-D-aThr-Val-Ala-Abu(2,3-dehydro)-OH	836.0	[145]

**Table 6:** Formula and molecular weight of depsipeptides inhibiting the growth of tumor cells.

Key	Depsipeptide	IUPAC Formula	Molecular Weight (g/mol)	Ref.
a	Skyllamycins	N-[I-3-[2-[(Z)-1-Propenyl]phenyl]propenoyl]-Cyclo[L-Thr*-L-Ala-[(3*S*)-3-Me-L-Asp-]-Gly-[(βS)-β-hydroxy-L-Phe-]-L-Pro-[(βS)-β-hydroxy-O-Me-L-Tyr-]-D-Trp-[(2*S*)-2-hydroxy-Gly-]-D-Leu-[(3*S*)-3-hydroxy-D-Leu-]-]	1483.6	[160]
b	Stereocalpin	Cyclo[Phe-N(Me)Phe-Unk]	492.6	[161]

**Table 7:** Formula and molecular weight of depsipeptides targeting topoisomerases in tumor cells.

Key	Depsipeptide	IUPAC Formula	Molecular Weight (g/mol)	Ref.
a	Fusaristatin A	3-[6,13-dimethyl-10-methylidene-2,5,9,12-tetraoxo-14-[(5*E*,7*E*)-3,7,11-trimethyl-4-oxoheptadeca-5,7-dienyl]-1-oxa-4,8,11-triazacyclotetradec-3-yl]propanamide	658.9	[170]
b	N-methylsansalvamide	Cyclo[Leu-Oleu-Val-N(Me)Leu-Phe]	600.8	[28]

**Table 8:** Formula and molecular weight of kahalalide F, the only depsipeptide inducing oncosis in tumor cells.

Depsipeptide	IUPAC Formula	Molecular Weight (g/mol)	Ref.
Kahalalide F	L-Val, N-(5-Me-1-oxohexyl)-D-valyl-l-threonyl-l-valyl-D-valyl-D-prolyl-l-ornithyl-D-alloisoleucyl-D-allothreonyl-D-alloisoleucyl-D-valyl-l-phenylalanyl-(2z)-2-amino-2-butenoyl-, (13->8)-lactone	1477.9	[180]

## Data Availability

Not applicable.
